# Evaluation of Safety and Protective Efficacy of a *waaJ* and *spiC* Double Deletion Korean Epidemic Strain of *Salmonella enterica* Serovar Gallinarum

**DOI:** 10.3389/fvets.2021.756123

**Published:** 2021-11-16

**Authors:** Jun-Feng Zhang, Ke Shang, Bai Wei, Yea-Jin Lee, Jong-Yeol Park, Hyung-Kwan Jang, Se-Yeoun Cha, Min Kang

**Affiliations:** Department of Veterinary Infectious Diseases and Avian Diseases, College of Veterinary Medicine and Center for Poultry Diseases Control, Jeonbuk National University, Iksan, South Korea

**Keywords:** DIVA, high attenuation, waaJ, *S. enterica* ser. Gallinarum, double deletion, spiC

## Abstract

With an aim to develop a highly attenuated and strongly immunogenic distinguishable vaccine candidate, a *waaJ* (a gene involved in the synthesis of lipopolysaccharide) and *spiC* (a virulence gene) double deletion Korean epidemic strain of *S. enterica* ser. Gallinarum (SG005) was constructed. Our results showed that the growth and biochemical characteristics were not altered by this double deletion. The double deletion strain contained dual markers. One was a bacteriological marker (rough phenotype) and the other was a serological marker helping distinguish infected chickens from vaccinated chickens. The double deletion strain showed good genetic stability and reduced resistance to environmental stresses *in vitro*; furthermore, it was extremely safe and highly avirulent in broilers. Single intramuscular or oral immunization of 7-day-old broilers with the double deletion strain could stimulate the body to produce antibody levels similar to the conventional vaccine strain SG9R. In addition, against a lethal wild-type challenge, it conferred effective protection that was comparable to that seen in the group vaccinated with SG9R. In conclusion, this double deletion strain may be an effective vaccine candidate for controlling *S. enterica* ser. Gallinarum infection in broilers.

## Introduction

*Salmonella enterica* serovar Gallinarum (*S. enterica* ser. Gallinarum) causes fowl typhoid (FT), an acute septicemic disease that occurs in chickens of all ages. Chicken infections are characterized by severe hepatosplenomegaly accompanied with bronze liver, anemia, and septicemia. The mortality and morbidity of FT can be as high as 80% (1). FT outbreaks have important economic significance in many areas, including Africa, Asia, the Middle East, and Central and South America (2). In Korea, *S. enterica* ser. Gallinarum was first officially reported during an FT outbreak in 1992 and is now found nationwide. FT has become one of the most prevalent bacterial diseases.

Many strategies have been used to prevent *S. enterica* ser. Gallinarum infection, including the establishment of strict biosecurity measures and the use of antibiotics and vaccines. The high costs of biosecurity measures limit their application in many developing countries, and long-term use of antibiotics can result in the emergence of multidrug-resistant strains. Vaccination seems to be the most effective strategy to control FT (1, 3). Until now, three major types of vaccines, namely inactivated vaccine, subunit vaccine, and live attenuated vaccine, have been developed to control *Salmonella* infections in the poultry industry. Inactivated and subunit vaccines can induce strong antibodies to eradicate extracellular bacteria; however, it is difficult for these antibodies to eliminate intracellular *Salmonella*; this goal can be achieved with the live attenuated strains of *Salmonella* (4). The live attenuated SG9R has been used as a commercial vaccine to control FT for nearly 60 years. However, some drawbacks, such as residual virulence in newly hatched chickens, insufficient protection, and vertical transmission, have been reported with its use (5, 6). Therefore, the development of a safe and effective *S. enterica* ser. Gallinarum vaccine remains a current research hotspot. Thus far, a number of deletion strains have been developed, and their protection efficiencies against FT have been reported (1, 7–14). However, none of the *S. enterica* ser. Gallinarum deletion strains have been found to have the feature to distinguish infected chickens from vaccinated chickens.

To construct a live attenuated *S. enterica* ser. Gallinarum vaccine with distinguishable capability, we firstly chose the *waaJ* gene. This gene codes for the lipopolysaccharide (LPS) 1,2-glucosyltransferase, which is involved in the synthesis of LPS (15). Deletion of the *waaJ* gene changes smooth LPS to rough LPS, which cannot react with antibodies against the O antigen. The *spiC* gene encodes an effector protein that is secreted by the *Salmonella* pathogenicity island-2 (SPI2) type III secretion system (T3SS), which plays important roles in *Salmonella* infections (16). Therefore, *spiC* was selected as the second target gene to be deleted.

In this study, we constructed a *waaJ* and *spiC* double deletion Korean epidemic strain of *S. enterica* ser. Gallinarum and evaluated its safety and protection efficacy for use as a live attenuated distinguishable vaccine candidate for the prevention of FT.

## Materials and Methods

### Bacterial Strains, Plasmids, Media, and Growth Conditions

A wild-type Korean epidemic virulent strain A17-DW-005 (SG005) of *S*. *enterica* ser. Gallinarum with resistance toward ciprofloxacin (CIP), streptomycin (STR), gentamicin (GEN), nalidixic acid (NAL), sulfisoxazole (FIS), and colistin (COL) was originally isolated from the liver of a 10-day-old broiler, which came from a broiler farm in Jeonbuk province in 2017. This strain was classified as type 1 by pulsed-field gel electrophoresis analysis (PFGE). Since type 1 has the highest proportion of all types, this strain was judged to be an epidemic strain. The suicide plasmid pRE112 and its host *Escherichia coli* (*E. coli*) χ7,213 were kept in our laboratory. pBluescript II SK (+) and pET-30 b (+) stored in our laboratory were used as intermediate vectors in this study. *E. coli* and *S. enterica* ser. Gallinarum were grown at 37°C in Luria–Bertani (LB) broth or on LB agar. When required, antibiotics were added to culture media at the following concentrations: ampicillin (Amp) at 100 μg/ml, kanamycin (Km) at 50 μg/ml, chloramphenicol at 25 μg/ml, and DL-α,ε-diaminopimelic acid (DAP) at 50 μg/ml. LB agar containing 5% sucrose was used for *sacB* gene-based counterselection in allelic exchange experiments. A rough attenuated *S. enterica* ser. Gallinarum live vaccine SG9R (Intervet International, Boxmeer, The Netherlands) was used as control vaccine in this study.

### Chickens

Ross broiler chickens were obtained from the YangJi Company (Cheonan, Choongnam, South Korea). Serum samples collected from all chickens in the present study were tested for *Salmonella* specific antibodies using a commercial ELISA kit (Biochek, cat# CK117, Crabethstraatt, Netherland). Moreover, the antibodies level of Avian influenza and Newcastle disease were detected by hemagglutination-inhibition (HI) test virus. The test results were all negative. 10% of the chickens were randomly selected and euthanized. The chickens were dissected to check whether there were any lesions in the internal organs. Liver and fecal samples were aseptically collected for routine *Salmonella* and avian pathogenic *Escherichia coli* isolation and culture, and the follow-up *Salmonella* infection experiment was performed after the complete negative was confirmed. The chickens were used after adaptation for a week. To ensure the best environmental conditions, the conditions of the isolator (temperature, humidity, and ventilation) were continuously monitored. The chickens were taken care of and handled by a well-trained staff.

### Ethics Statement

All experimental and animal management procedures were undertaken in accordance with the requirements of the Animal Care and Ethics Committee of Jeonbuk National University. The animal facility at Jeonbuk National University is fully accredited by the National Association of Laboratory Animal Care (approval number: JBNU 2020-0162).

### Construction of Deletion Strains of *S. enterica* Ser. Gallinarum by Allelic Exchange

Construction of deletion strains was performed by the allelic exchange method using the suicide vector pRE112 as described previously (Supplementary Figure 1) (17). The 1,000-bp upstream fragment of the *waaJ* gene was amplified from the genomic DNA of *S. enterica* ser. Gallinarum using a pair of primers (W1 and W2) by PCR (Supplementary Table 1). The PCR product was cloned into the *Xba*I and *Bam*HI sites of the pBluescript II SK(+) vector, resulting in pSK-*waaJ*-up. The 1,000-bp downstream fragment of the *waaJ* gene was then PCR-amplified using a pair of primers (W3 and W4) and cloned into the *Bam*HI and *Kpn*I sites of pSK-*waaJ*-up to obtain pSKΔ*waaJ*. The 2,000-bp fragment, which included the upstream and downstream fragments of the *waaJ* gene from the *Xba*I- and *Kpn*I-digested plasmid pSKΔ*waaJ*, was ligated into plasmid pRE112 to yield the suicide plasmid pREΔ*waaJ*. The transfer of recombinant suicide plasmids into SG005 was accomplished by conjugation using *E. coli* χ7213 (pREΔ*waaJ*) as the plasmid donor. Strains containing single-crossover plasmid insertions (SG005*waaJ*::pREΔ*waaJ*) were isolated on plates containing chloramphenicol. The first homologous recombination was confirmed with the primers W5 and W6. A loss of the suicide vector after the second recombination between homologous regions (i.e., allelic exchange) was selected for by using the *sacB*-based sucrose sensitivity counterselection system. The second homologous recombination was confirmed with the primers W6 and W7. Primers W8 and W9 were used to confirm whether the full *waaJ* gene was removed from the genome. Primers W10 and W11 were used to confirm whether the suicide plasmid was removed from the genome (Supplementary Figure 2). This deletion strain was designated as SG005Δ*waaJ*. Using the same method, deletion of *spiC* was introduced into SG005, and this deletion strain was designated as SG005Δ*spiC*. Notably, in the construction of the suicide plasmid pREΔ*spiC*, pET-30 b (+) was used as an intermediate vector. Similarly, the deletion of *spiC* was introduced into SG005Δ*waaJ*, and the double deletion strain was designated as SG005Δ*waaJ*Δ*spiC*.

The complemented strains were constructed previously described with some modifications (18–20). The gene *waaJ* was PCR-amplified from the wild type strain SG005 using the primers pBR322-*waaJ*-F and pBR322-*waaJ*-R (Supplementary Table 1), which were designed based on the coding sequence of *waaJ* gene. After amplification, the DNA fragment was digested by *Bam*HI and *Sal*I and ligated to the plasmid pBR322. The resulting vector pBR322-*waaJ* was introduced into SG005Δ*waaJ* by electroporation to produce the complemented strain SG005Δ*waaJ* (pBR322-*waaJ*). Electroporation on the electroporator (Bio-Rad Laboratories, Hercules, CA, USA) was performed using optimized parameters (2.5 kV, 200 Ω, 25 μF, 5 ms). Similarly, the complemented strains SG005Δ*spiC* (pBR322-*spiC*) and SG005Δ*waaJ*Δ*spiC* (pBR322*-waaJ*-*spiC*) were constructed. It should be pointed out that when constructing SG005Δ*waaJ*Δ*spiC* (pBR322-*waaJ*-*spiC*), the *waaJ* gene and *spiC* gene were inserted into the plasmid pBR322 in tandem.

### Analysis of *in vitro* Growth and Biochemical Characteristics

For the *in vitro* growth analysis of SG005, SG005Δ*waaJ*, SG005Δ*spiC*, and SG005Δ*waaJ*Δ*spiC*, a single colony of each strain was inoculated into 5 ml of LB broth and cultured at 37°C with shaking at 200 rpm for approximately 15 h. Subsequently, the optical density (OD_590_) of each strain was measured by a Genesys 10S UV-Vis spectrophotometer (Thermo Fisher Scientific, Waltham, USA), and cultures were diluted in 20 ml of LB broth; then, the OD_590_ was determined to achieve an initial concentration (OD_590_ = 0.03) at the starting time point (0 h). The cultures were incubated at 37°C with shaking at 200 rpm, and the OD_590_ was determined at 1, 2, 3, 4, 5, 6, 7, and 8 h. Each strain was tested in triplicate in three independent experiments. The biochemical properties of the strains were tested according to the API20E handbook (Biomerieux, SA, France).

### Phenotype Identification

O9 antigen is one of the antigens produced on *S. enterica* ser. Gallinarum. *S. enterica* ser. Gallinarum with smooth phenotype have O9 antigen and therefore can be agglutinated by O9 antibody. Briefly, colonies from LB agar plates were mixed with 20 μl of phosphate-buffered saline (PBS) and 20 μl of the O9 factor rabbit antiserum (SSI^®^ SALMONELLA ANTISERA, Denmark) on a glass slide. Mixtures were rotated for 1 min and observed for agglutination. The acriflavine agglutination test was performed to detect the rough phenotype characteristic of the deletion strains (21). Briefly, colonies of a 24-h culture prepared from the deletion strains on LB agar plates were mixed with 20 μl of 0.2% acriflavine on a glass slide. Mixtures were rotated for 1 min and observed for agglutination.

### Auto-Aggregation Assay

Auto-aggregation refers to the phenomenon that bacteria aggregate into clusters by themselves during the cultivation process. The stronger is the hydrophobicity of the cell surface, the higher is the auto-aggregation ability of the cell. The auto-aggregation assay was performed based on a previously described method with some modifications (22). Briefly, SG005, SG005Δ*waaJ*, SG005Δ*spiC*, and SG005Δ*waaJ*Δ*spiC* were statically grown in 5 ml of LB broth at 37°C for 24 h in 15-ml conical tubes. The upper 0.5 ml was carefully removed to measure its OD_590_ (recorded as OD_590__prevortex_). Then, the remaining culture in the tube was mixed by vortexing to resuspend the aggregated cells, and 0.5 ml of the suspension was removed, and its OD_590_ was measured (recorded as OD_590_ postvortex). The “percent aggregation” was calculated using the formula: 100% × (OD_590__postvortex_ – OD_590__prevortex_)/OD_590__postvortex_.

### Stability Analysis

To determine the stability of the deletion strains, the liquid culture of each strain was continuously passaged 60 times every 12 h. Every 10 generations, DNA was extracted for PCR identification.

### Environmental Safety Evaluation

The sensitivity of SG005, SG005Δ*waaJ*, SG005Δ*spiC*, and SG005Δ*waaJ*Δ*spiC* to ultraviolet (UV) light and various disinfection agents was evaluated as described with some modifications (23, 24). Bacteria were grown to OD_590_ = 1.0 at 37°C with shaking at 200 rpm and were diluted in PBS to 10^9^ colony forming units (CFU)/ml. One milliliter of suspension was then transferred to a sterile Petri dish and exposed to UV radiation (Sankyo denki UV G30TB: 30 W, distance: 45 cm) for 1 min. For testing oxidative stress and alkali and acid tolerance, each bacterial suspension and the stressor were mixed in equal volume and incubated at room temperature (RT, 25°C): 100-μM hydrogen peroxide (H_2_O_2_) for 5 min, 12.5-mM sodium hydroxide (NaOH) for 5 min, and 50-mM citric acid for 20 min. Finally, the number of viable bacteria was determined by plating serial dilutions onto LB agar plates. The experiment was performed three times.

### Assessment of LD_50_

To determine the 50% lethal dose (LD_50_), SG005, SG005Δ*waaJ*, SG005Δ*spiC*, and SG005Δ*waaJ*Δ*spiC* were grown for 12 h at 37°C in LB broth. One milliliter of broth from each grown bacterium was inoculated into 100 ml of LB broth. After the inoculated strains were grown to OD_590_ = 1.0, the bacterial cells were recovered by centrifugation for 10 min at 2,400 g at RT. The harvested cells were suspended in PBS to a concentration of 10^11^-10^12^ CFU/ml. The suspended strains were serially diluted by 10-fold. The 7-day-old chickens were intramuscularly (IM) injected or orally inoculated with each dose of these strains (10^1^-10^11^ CFU). Six chickens were used for each dose. Feed and water were not provided to the tested chickens for 4 h before and until 1 h after the treatment. The treated chickens were observed twice a day for 2 weeks after administration. Clinical scores were determined and recorded using a system as we have previously described (25). Briefly, disease severity was scored as follows: 0, normal; 1, depression and ruffled feathers; 2, depression, ruffled feathers, and respiratory distress; 3, abovementioned clinical signs plus anorexia, emaciation, and green-yellowish diarrhea; and 4, death. When the birds showed a clinical score of 3 (humane endpoint), they were euthanized immediately by cervical dislocation performed by trained veterinarians. LD_50_ was calculated by the method of Reed and Muench (26).

### Organ Colonization and Persistence

Bacterial persistence and clearance were analyzed by counting the number of the bacteria per gram of liver or spleen. Seven-day-old chickens were randomly divided into four groups (*n* = 12 for each group): SG005Δ*waaJ* group, SG005Δ*spiC* group, SG005Δ*waaJ*Δ*spiC* group, and PBS control group. The first three groups were injected intramuscularly with 1 × 10^8^ CFU of SG005Δ*waaJ*, SG005Δ*spiC*, and SG005Δ*waaJ*Δ*spiC*, respectively. The last group was inoculated with an equal volume of PBS in the same way. Three chickens from each group were euthanized at 2, 6, 10, and 14 days post-inoculation (dpi), and liver and spleen samples were aseptically removed. The samples were weighed and suspended in 1 ml of PBS and then individually homogenized. The homogenates were diluted serially and subsequently plated on MacConkey agar (Difco Laboratories, Detroit, MI, USA) at 37°C. After overnight cultivation, the bacterial number was counted.

### Vaccination Experiment

Sixty 7-day-old chickens were divided into five groups (*n* = 12 for each group) to evaluate the protective efficacy of the deletion strains. The group 1 (PBS negative control) and group 2 (PBS positive control) were intramuscularly inoculated with 500 μl of PBS (pH 7.2). The group 3 and group 4 were intramuscularly inoculated with 10^8^ CFU of SG9R and SG005Δ*waaJ*Δ*spiC*, respectively. The group 5 was orally inoculated with 10^8^ CFU of SG005Δ*waaJ*Δ*spiC*. After 4 weeks, except for the group 1, chickens in other groups were challenged intramuscularly with 10^8^ CFU of virulent SG005. Deaths and clinical signs were recorded daily for 2 weeks. At the end of this period, all surviving chickens were euthanized. Clinical scores were determined and recorded following the method previously described (25).

### Antibody Production Assay

A laboratory-made indirect enzyme-linked immunosorbent assay (ELISA) using heat-killed whole *S*. *enterica* ser. Gallinarum bacteria as the coating antigen was applied to quantify IgG in the serum. Briefly, bacterial antigen (10^6^ CFU/well) was coated in a 96-well plate and incubated overnight at 4°C. After blocking, the sera samples collected on days 14, 21, and 28 were diluted (1:400) with 2% bovine serum albumin (BSA) and added to the wells. The wells were incubated with a 1:8,000 dilution of affinity-purified, peroxidase-labeled goat anti-chicken IgG (H+L) (KPL Inc., MD, USA) for 1 h. OD_450_ was measured with a microplate reader VICTORTM X4 (PerkinElmer Inc., Waltham, MA, USA) after the reaction was stopped with 4.5 N H_2_SO_4_. The OD_450_ average value of negative serum samples plus the standard deviation (SD) of three times was used as the cut-off value (27).

### Capability of DIVA (Differentiation of Infected and Vaccinated Animals) Based Serodiagnosis

The serum plate agglutination (SPA) test was used to assess the DIVA capability of SG005Δ*waaJ*, SG005Δ*spiC*, and SG005Δ*waaJ*Δ*spiC*. Seven-day-old chickens were intramuscularly inoculated with each deletion strain. The sera were prepared on 14, 21, and 28 dpi by collecting blood from wing veins. The blood was allowed to coagulate for 1 h at RT, centrifuged for 10 min at 600 g, and then, the clean phase of serum was collected. Serum samples (30 μl) were mixed with an equal volume of *S. enterica* ser. Pullorum/Gallinarum standard antigen (Green Cross Veterinary Products, Yongin, South Korea), and the agglutination reaction was observed.

### Statistical Analysis

Statistical analysis was performed using SPSS version 21.0 (SPSS Inc., Chicago, IL, USA). The data were analyzed by one-way analysis of variance (ANOVA). Differences were considered statistically significant at: ^*^P < 0.05, ^**^P < 0.01, ^***^P < 0.001.

## Results

### Constructions of *S. enterica* Ser. Gallinarum Deletion Strains

The deletion of the *waaJ* gene in SG005 was confirmed by PCR using the primers W6 and W7 with the expected amplicon sizes of 2,490 and 1,476 bp for the wild type and deletion type of the *waaJ* gene, respectively. The deletion of the *spiC* gene in SG005 was confirmed by PCR using the primers S6 and S7 with the expected amplicon sizes of 2,003 and 1,619 bp for the wild type and deletion type of the *spiC* gene, respectively (Supplementary Figure 3). In the three deletion strains, the corresponding target gene and suicide plasmid could not be detected by PCR (Supplementary Figure 4). The sequencing results also showed that the deletion strains SG005Δ*waaJ*, SG005Δ*spiC*, and SG005Δ*waaJ*Δ*spiC* were constructed successfully (Supplementary Text 1).

### Growth and Biochemical Characteristics

**Figure 1:**
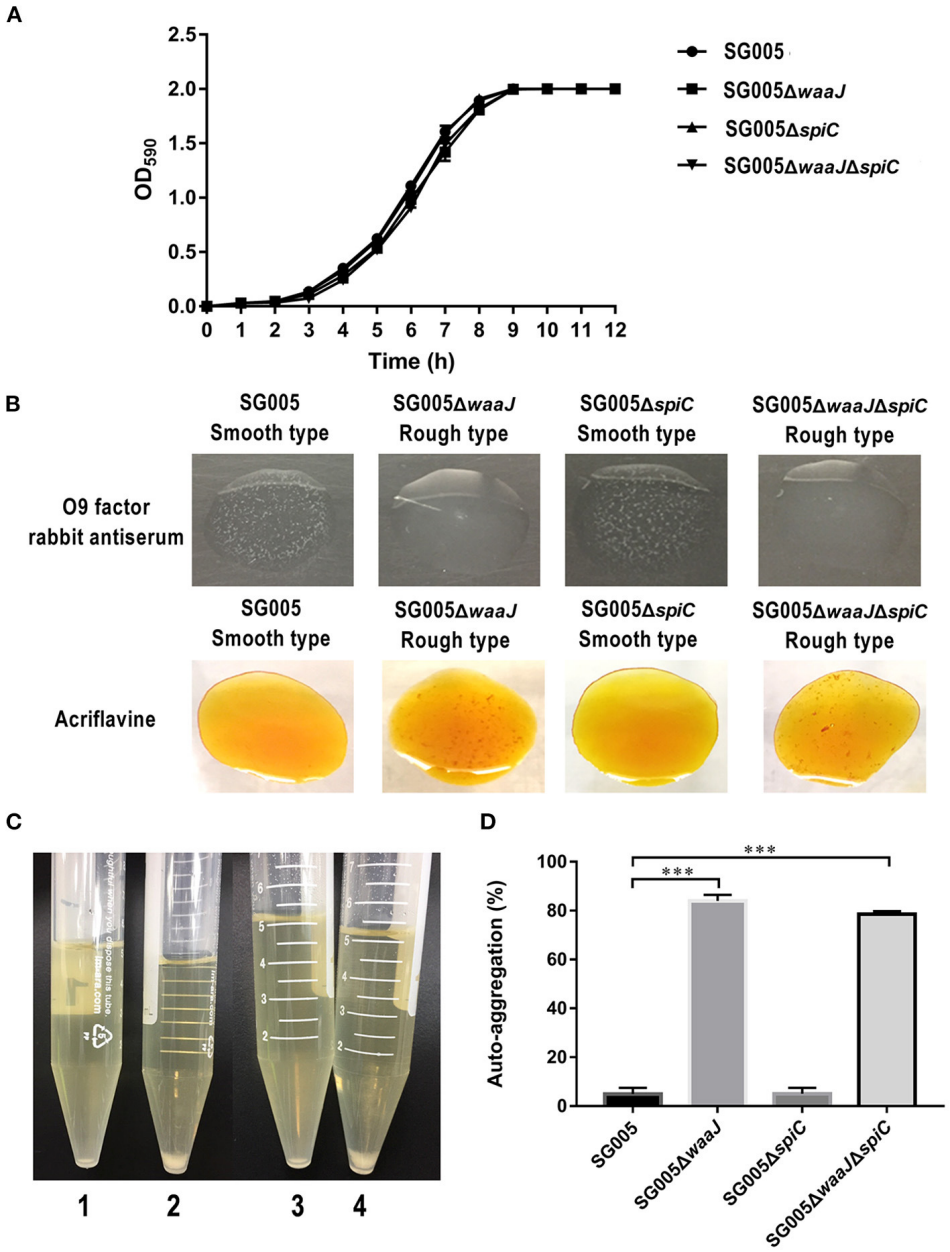
Growth curves, phenotypic characterization, and auto-aggregation assay. **(A)** Growth curves of wild-type SG005 and the deletion strains. SG005, SG005Δ*waaJ*, SG005Δ*spiC*, and SG005Δ*waaJ*Δ*spiC* showed a similar growth curve in LB broth. **(B)** Phenotype identification. Agglutination was examined with O9 factor rabbit antiserum and acriflavine. Pictures were taken within 3 min. **(C)** Visual auto-aggregation of SG005 (1), SG005Δ*waaJ* (2), SG005Δ*spiC* (3), and SG005Δ*waaJ*Δ*spiC* (4). **(D)** Auto-aggregation (%). Bacterial cultures were statically grown in LB broth for 24 h at 37°C. Statistical analysis was done using one-way ANOVA. ***P < 0.001. Each experiment was repeated three times.

Growth curve analysis revealed no significant differences between the wild type and each deletion strain when cultured in LB broth at 37°C (Figure 1A). Results of biochemical tests including 2-nitrophenyl-β D-galactopyranoside, L-arginine, L-lysine, L-ornithine, trisodium citrate, sodium thiosulfate, urea, L-tryptophane, L-tryptophane, sodium pyruvate, gelatin, D-glucose, D-mannitol, inositol, D-sorbitol, L-rhamnose, D-sucrose, D-melibiose, amygdalin, and L-arabinose were the same between wild type and each deletion strain, suggesting that deletions in these two genes did not alter the biochemical characteristics of SG005 (Supplementary Table 2).

### Phenotype Identification

SG005 and SG005Δ*spiC* were agglutinated with O9 factor rabbit antiserum but not with acriflavine, indicating that they had a smooth phenotype. However, SG005Δ*waaJ* and SG005Δ*waaJ*Δ*spiC* were not agglutinated with O9 factor rabbit antiserum but were agglutinated with acriflavine, indicating that they had a rough phenotype (Figure 1B). The complemented strains SG005Δ*waaJ* (pBR322-*waaJ*), SG005Δ*spiC* (pBR322-*spiC*) and SG005Δ*waaJ*Δ*spiC* (pBR322-*waaJ-spiC*) were all agglutinated with O9 factor rabbit antiserum but not with acriflavine. Therefore, complementary analysis proved that the deletion of the *waaJ* gene was the direct cause of the bacteria changing from smooth to rough.

### Auto-Aggregation Assay

Auto-aggregation was tested in LB broth (Figure 1C), and the aggregation percent was calculated using OD_590_ measurements from these cultures. Both SG005 and SG005Δ*spiC* showed ~5% aggregation, whereas SG005Δ*waaJ* and SG005Δ*waaJ*Δ*spiC* demonstrated 84 and 79% aggregation, respectively (Figure 1D).

### Stability

**Figure 2:**
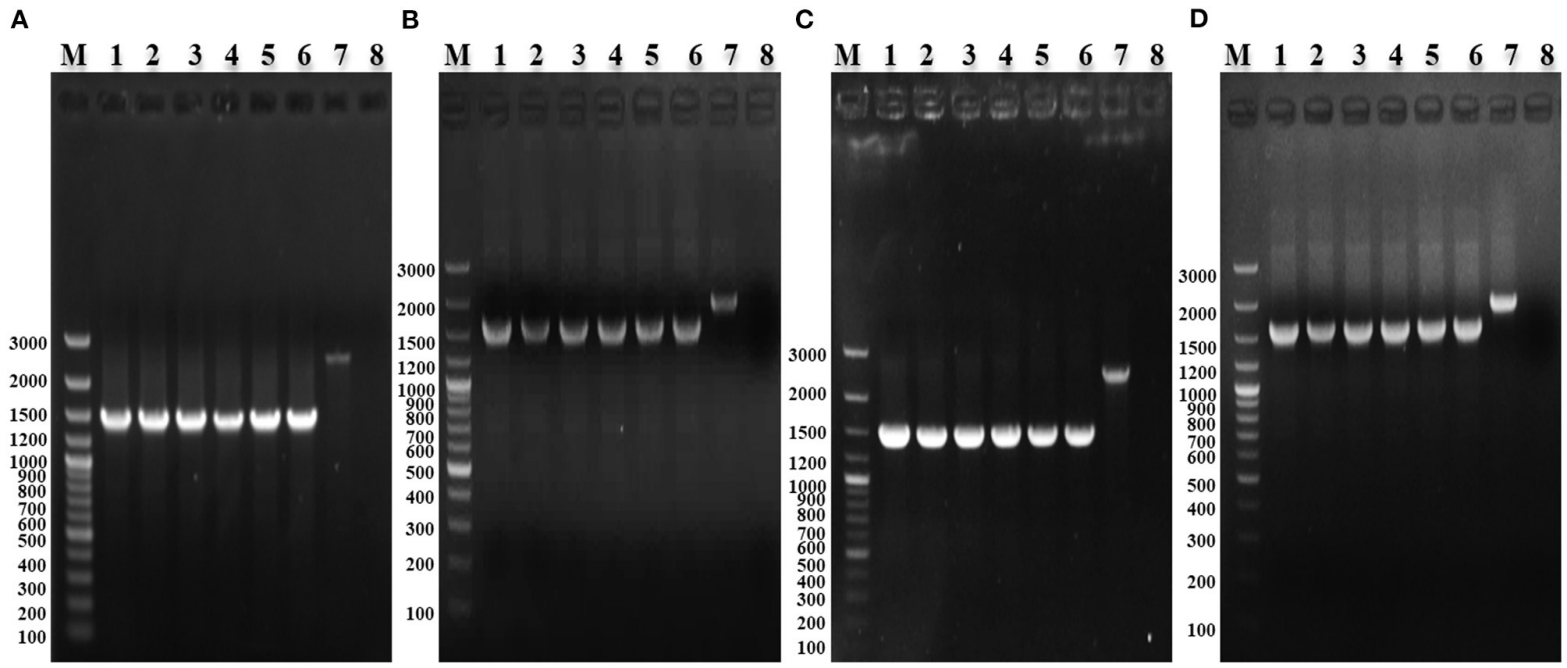
PCR identification of the stability of *S. enterica* ser. Gallinarum deletion strains. **(A)** PCR identification of SG005Δ*waaJ* with W6/W7. M: GeneRuler 100 bp Plus DNA Ladder (Thermo Fisher Scientific, Waltham, MA, USA); 1–6: PCR-amplified products from the 10th, 20th, 30th, 40th, 50th, and 60th passages of SG005Δ*waaJ*; 7: Wild-type SG005; and 8: Negative control. **(B)** PCR identification of SG005Δ*spiC* with S6/S7. M: GeneRuler 100 bp Plus DNA Ladder; 1–6: PCR-amplified products from the 10th, 20th, 30th, 40th, 50th, and 60th passages of SG005Δ*spiC*; 7: Wild-type SG005; and 8: Negative control. **(C)** PCR identification of SG005Δ*waaJ*Δ*spiC* with W6/W7. M: GeneRuler 100 bp Plus DNA Ladder; 1–6: PCR-amplified products from the 10th, 20th, 30th, 40th, 50th, and 60th passages of SG005Δ*waaJ*Δ*spiC*; 7: Wild-type SG005; and 8: Negative control. **(D)** PCR identification of SG005Δ*waaJ*Δ*spiC* with S6/S7. M: GeneRuler 100 bp Plus DNA Ladder; 1–6: PCR-amplified products from the 10th, 20th, 30th, 40th, 50th, and 60th passages of SG005Δ*waaJ*Δ*spiC*; 7: Wild-type SG005; and 8: Negative control.

The deletion Strains were serially passaged 60 times in LB medium, and the absence of *waaJ* and *spiC* genes was confirmed by PCR, indicating that each deletion strain had good genetic stability (Figure 2).

### Bacterial Resistance to Environmental Stress

**Figure 3:**
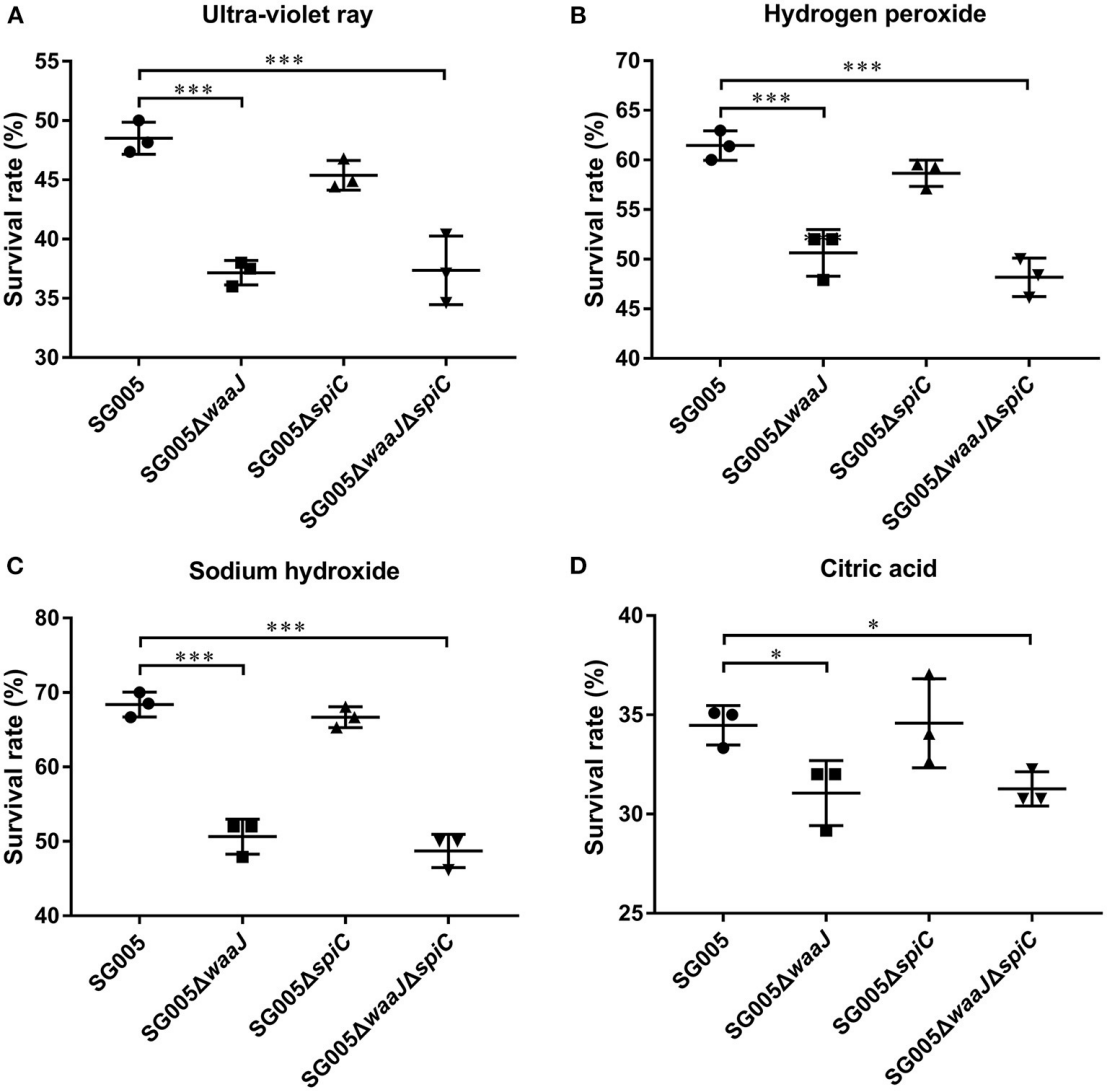
Survival rates of SG005 and deletion strains in different environmental stresses. **(A)** UV irradiation for 1 min. **(B)** Hydrogen peroxide (100 μM for 5 min). **(C)** Sodium hydroxide (12.5 mM for 5 min). **(D)** Citric acid (50 mM for 20 min). Statistical analysis was done using one-way ANOVA. *P < 0.05, **P < 0.01, ***P < 0.001. Each experiment was repeated three times.

After UV irradiation, the survival rates for SG005, SG005Δ*waaJ*, SG005Δ*spiC*, and SG005Δ*waaJ*Δ*spiC* were 48, 37, 45, and 37%, respectively (Figure 3A). A significant difference (*P* < 0.001) was seen between Δ*waaJ* strains (SG005Δ*waaJ* and SG005Δ*waaJ*Δ*spiC*) and non-Δ*waaJ* strains (SG005 and SG005Δ*spiC*), indicating that the deletion of the *waaJ* gene increased the sensitivity of the strain to UV. In terms of oxidative stress, SG005Δ*waaJ* and SG005Δ*waaJ*Δ*spiC* with survival rates of 52 and 50% showed reduced resistance compared to SG005 and SG005Δ*spiC* with survival rates of 59 and 60% (*P* < 0.001) in H_2_O_2_ (Figure 3B). For alkali endurance, the survival rates for SG005, SG005Δ*waaJ*, SG005Δ*spiC*, and SG005Δ*waaJ*Δ*spiC* were 68, 51, 67, and 49%, respectively. A significant difference was noted between Δ*waaJ* strains and non-Δ*waaJ* strains (*P* < 0.001; Figure 3C). For acid endurance, the survival rates for SG005, SG005Δ*waaJ*, SG005Δ*spiC*, and SG005Δ*waaJ*Δ*spiC* were 34, 31, 35, and 31%, respectively. Statistical analysis revealed a difference between Δ*waaJ* strains and non-Δ*waaJ* strains (*P* < 0.05; Figure 3D). However, deletion of *spiC* gene had no effect on bacterial resistance to these environmental stresses.

### Determination of LD_50_

To confirm the safety of the deletion strains, the virulence of wild and deletion strains was evaluated in 7-day-old chickens. The IM LD_50_ of SG005Δ*waaJ* and SG005Δ*spiC* was about 10^8^-fold higher than that of the wild strain. The IM LD_50_ of SG005Δ*waaJ*Δ*spiC* was about 10^10^-fold higher than that of the wild strain. The oral LD_50_ of the three deletion strains SG005Δ*waaJ*, SG005Δ*spiC*, and SG005Δ*waaJ*Δ*spiC* was about 10^5^-fold higher than that of the wild strain SG005 (Table 1).

**Table 1:** Chicken LD_50_ for the wild-type SG005 and deletion strains.

**Strain**	**LD_50_^a^(CFU)**	
	**IM^b^**	**Oral^c^**
SG005	<10^1^	1.6 × 10^5^
SG005Δ*waaJ*	1.1 × 10^9^	>10^10^
SG005Δ*spiC*	5.7 × 10^9^	>10^10^
SG005Δ*waaJ*Δ*spiC*	>10^11^	>10^11^

a *LD_50_: 50% lethal dose.*

b *Chickens were inoculated intramuscularly (IM) with SG005 and the deletion strains.*

c *Chickens were inoculated orally with SG005 and the deletion strains*.

### Organ Colonization and Persistence

**Figure 4:**
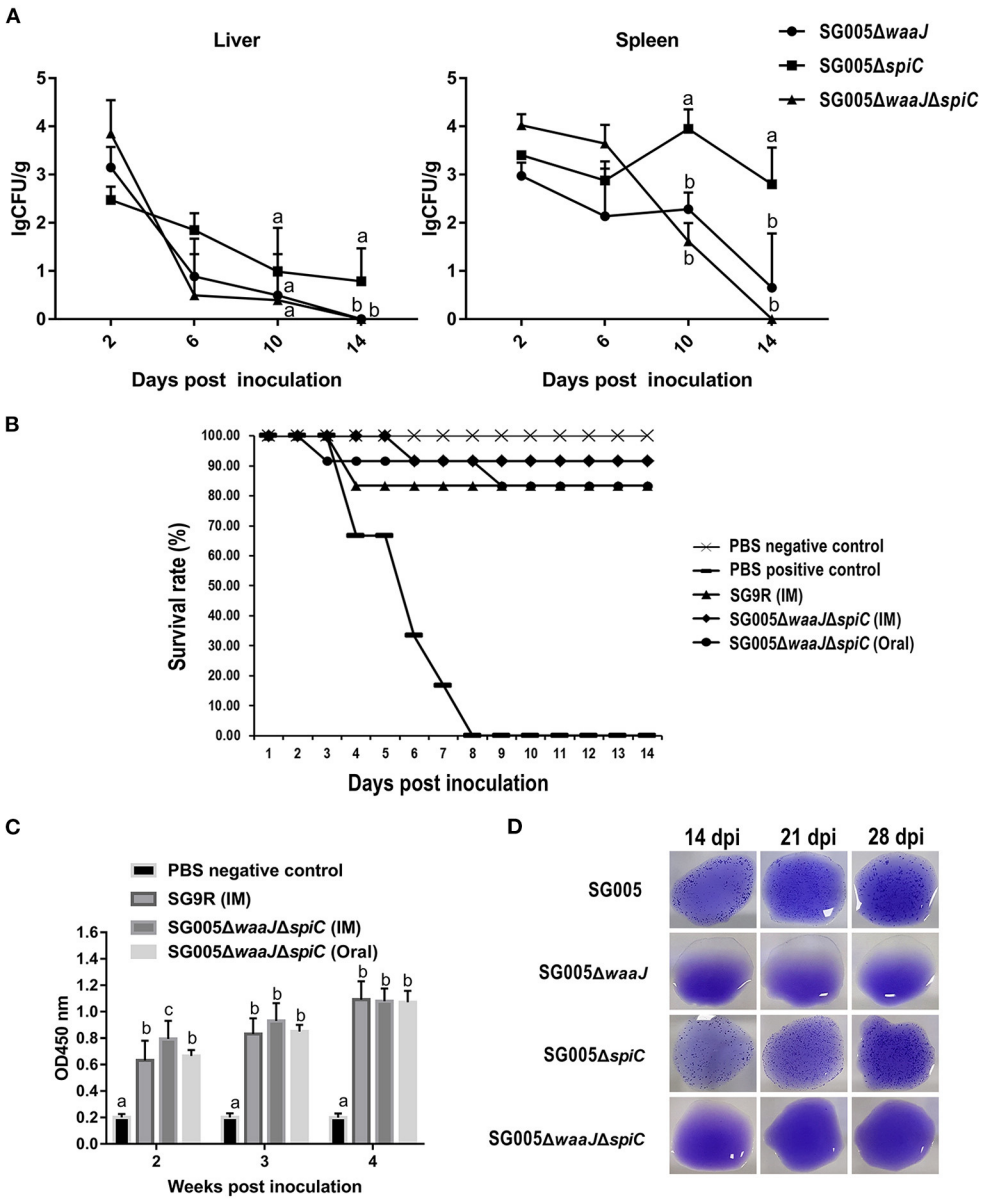
Organ colonization, protective efficacy, humoral immune response, and DIVA capability analysis. **(A)** Colonization and persistence of the deletion strains in the liver and spleen after inoculation of 7-day-old chickens with 10^8^ CFU bacterial cells. Values were represented as lgCFU/g sample. All broilers inoculated with SG005 died within 3 days, and the number of bacteria isolated from the liver and spleen was about 8 lgCFU/g. **(B)** Protective ability of the deletion strains against the virulent SG005 challenge. At 14 dpi, chickens from each group were challenged intramuscularly with 1 × 10^8^ CFU wild-type SG005. Protection was expressed by percentage survival after challenge. SG9R was used as a vaccine control. **(C)** Levels of humoral immune responses in vaccinated vs. control chickens. Serum IgG antibody responses against the deletion strains were measured by indirect ELISA. OD_450_, optical density at 450 nm. The OD_450_ average value of negative serum samples plus the standard deviation (SD) of three times was used as the cut-off value, which was found to be 0.281. **(D)** Capability of DIVA based on a serum plate agglutination test. Sera collected on 14, 21, and 28 dpi were tested for reactivity to the *S. enterica* ser. Pullorum/Gallinarum standard antigen. Statistical analysis was done using one-way ANOVA. Different letters mean significant differences between groups (*P* < 0.05).

The results of bacteria persistence and clearance in organs were shown in Figure 4A. SG005Δ*waaJ*Δ*spiC* colonized and persisted in the liver and spleen for at least 10 days. Within these 10 days, the number of SG005Δ*waaJ*Δ*spiC* gradually decreased. At 14 dpi, SG005Δ*waaJ*Δ*spiC* could not be isolated from the liver and spleen. However, SG005Δ*waaJ* and SG005Δ*spiC* could still be isolated, indicating that the double deletion strain could be cleared more quickly than the single deletion strains.

### Protective Efficacy Against Virulent *S. enterica* Ser. Gallinarum Challenge

Chickens that were intramuscularly inoculated with 10^8^ CFU of SG9R and SG005Δ*waaJ*Δ*spiC* showed 83 and 92% protection against *S. enterica* ser. Gallinarum challenge, respectively. Chickens that were orally inoculated with 10^8^ CFU of SG005Δ*waaJ*Δ*spiC* showed 83% protection. All chickens died in the PBS-positive control group (Figure 4B). Depression, anorexia, and diarrhea were observed in the PBS-positive control group, whereas no clinical signs were observed in the SG005Δ*waaJ*Δ*spiC* group.

### Humoral Immune Response Generated by the Deletion Strains

Statistical analysis showed a significant difference in the humoral immune response in chickens immunized with the deletion strains compared with the PBS control group. Serum IgG levels at each post-immunization time point were significantly higher in the immunized groups than in the PBS control group. The IgG level of the SG005Δ*waaJ*Δ*spiC* group immunized by intramuscular injection oral administration was not significantly different from that of the SG9R group at 3 and 4 weeks after inoculation (*P* > 0.05; Figure 4C).

### Differentiation of Serum Antibodies to *S. enterica* Ser. Gallinarum

Antibodies to *S. enterica* ser. Gallinarum were detected by the SPA test using the SG005 antigen. Sera collected from SG005Δ*waaJ-* or SG005Δ*waaJ*Δ*spiC*-immunized chickens at different time intervals were identified as SPA-negative (Figure 4D). However, sera collected from SG005- or SG005Δ*spiC*-immunized chickens were identified as SPA-positive. Overall, SG005Δ*waaJ* and SG005Δ*waaJ*Δ*spiC* showed evident distinguishable capability.

## Discussion

Many shortcomings of SG9R vaccine limit its application. The residual virulence of SG9R caused liver and spleen lesions in day-old broilers and brown layers, and a single point mutation of the *waaJ* gene can make SG9R lose DIVA capability. Moreover, SG9R has the potential to revert to a more virulent phenotype in the field (28).

Our goal was to develop a highly attenuated and strongly immunogenic distinguishable vaccine. The reasons we decided to delete *waaJ* were as follows: Firstly, the protein encoded by the *waaJ* gene is involved in the synthesis of LPS. LPS is a major virulence factor of *S. enterica* ser. Typhimurium and is composed of lipid A, core oligosaccharide (C-OS), and O-antigen polysaccharide (O-PS). Partial deletion of LPS will directly reduce bacterial virulence (29). Secondly, deletion of the *waaJ* (*rfaJ*) gene can be used as a DIVA marker in current *Salmonella* serosurveillance programmes based on the detection of antibodies against LPS of *Salmonella* (30). Thirdly, the deletion of *waaJ* gene does not affect the protective capacities of the strain. It was reported that applying deletions in the *waaJ* gene in *S. enterica* ser. Typhimurium strain 112910a allows differentiation of infected and vaccinated pigs in an LPS based ELISA without reducing the strain's protective capacities in mice (30). Therefore, the *waaJ* gene is a good choice for constructing a distinguishable vaccine. Reportedly, the virulence of a *spiC* deletion strain of *S. enterica* ser. Pullorum was significantly reduced, and this deletion strain could induce high levels of circulating antibody and protective immunity in chickens (31). It has also been reported that a *spiC* deletion strain of *S. enterica* ser. Typhimurium was unable to survive within macrophages and had greatly reduced virulence in mice (32). Moreover, a *S. enterica* ser. Pullorum vaccine candidate S06004Δ*spiC*Δ*waaL* (a gene also related to LPS synthesis) with DIVA capability has been reported. Therefore, the virulence gene *spiC* was selected as the second gene to be deleted.

Here, there was another point that needed to be explained, that is why we did not choose *waaL* gene, but *waaJ* gene. Compared with the *waaL* gene, the reasons for choosing to delete the *waaJ* gene were as follows: Firstly, *waaL* gene codes a membrane enzyme implicated in ligating the O-antigen to the lipid A-core oligosaccharide. The *waaJ* deletion strain deleted more LPS fragment than the *waaL* deletion strain. The knockout part of the *waaJ* deletion strain contained the knockout part of the *waaL* deletion strain (33). Therefore, we speculated that compared with the *waaL* gene, deleting the *waaJ* gene may reduce the virulence more significantly and the DIVA effect in *waaJ* deletion strain may be more obvious than that of *waaL* deletion strain. Secondly, through whole-genome sequencing, it was found that there was a point mutation in the *waaJ* gene of the commercial vaccine strain, which prevented the protein encoded by *waaJ* from being synthesized. LPS defect caused by a point mutation in the *waaJ* gene may be one of the major mechanisms of vaccine strain attenuation (28). The commercial vaccine has been used for decades, and its immunogenicity is widely recognized by the poultry industry. Therefore, this reminded us that deleting the *waaJ* gene may be a good choice when developing new vaccines.

The growth and biochemical properties were not altered by deletions of *waaJ* or *spiC* or both genes from *S. enterica* ser. Gallinarum, suggesting that both genes may not be related to the bacterial metabolic pathway (Figure 1A and Supplementary Table 2). *S. enterica* ser. Gallinarum with smooth phenotype has O9 antigen and can therefore be agglutinated by O9 antibody. Our result showed that SG005 and SG005Δ*spiC* were agglutinated with O9 factor rabbit antiserum but SG005Δ*waaJ* and SG005Δ*waaJ*Δ*spiC* were not, indicating that the *waaJ* deletion strain did not contain O9 antigen. We performed the acriflavine agglutination test, which is used to detect the rough phenotype of bacteria (21). Our results were consistent with our expectations. SG005Δ*waaJ* and SG005Δ*waaJ*Δ*spiC* were agglutinated with acriflavine, whereas SG005 and SG00Δ*spiC* were not, indicating that deletion of the *waaJ* gene directly caused the LPS to change from smooth to rough (Figure 1B). In addition, both SG005Δ*waaJ* and SG005Δ*waaJ*Δ*spiC* showed evident auto-agglutination (Figures 1C,D). This is because the increased hydrophobicity of the rough *Salmonella* surface makes the cells aggregate and settle. In brief, the deletion of *waaJ* and *spiC* genes had no effect on the growth and biochemical characteristics of the bacteria, and the deletion of the *waaJ* gene not only changed the phenotype of the bacteria from a smooth type to a rough type but also made the bacteria exhibit obvious auto-aggregation.

The sensitivity of strains to different environmental stresses is an important indicator for testing the environmental safety of vaccines. As an important component of LPS, the O antigen structure may prevent antibacterial agents, such as disinfectants, from reaching the target sites in the cell wall to some extent, thereby improving its environmental resistance. The destruction of the LPS structure could reportedly reduce the resistance of bacteria to environmental stresses (24). In our study, the *waaJ* deletion strains (SG005Δ*waaJ* and SG005Δ*waaJ*Δ*spiC*) showed reduced resistance to UV, oxidative stress, and alkalis and acids, suggesting that the integrity of LPS is instrumental in the resistance of bacteria to environmental stresses (Figure 3). Similar results have been reported for *S. enterica* ser. Pullorum, *S. enterica* ser. Enteritidis, and *E. coli* (21, 24, 34). These results indicated that the deletion of the *waaJ* gene significantly increased the sensitivity of bacteria to environmental stress.

The deletion of the *waaJ* gene was reported to not only confer the DIVA capability to the smooth type strains but also reduce the virulence. The deletion of the *waaJ* gene consequently obstructed the expression of a part of the outer core and the O antigen of LPS. LPS is an important virulence factor, and partial deletion of LPS will directly reduce bacterial virulence. It has been reported that *S. enterica* ser. Typhimurium deletion strain 11310 (*waaJ44*) was significantly attenuated (about 10^5^-fold) in mice (29). *S. enterica* ser. Enteritidis deletion strain SEM1C3Δ*waaJ* had a LD_50_ at least 100 times higher than that of the parental strain in chickens (35). The *spiC* gene is closely related to the virulence of bacteria. It has been reported that the LD_50_ of *S. enterica* ser. Pullorum S06004Δ*spiC* was 200 times higher than that of the parent strain (23). Furthermore, the LD_50_ of *S. enterica* ser. Enteritidis C50041Δ*spiC* was reportedly 900 times higher than that of the wild-type strain (16). The LD_50_ of *S. enterica* ser. TyphimuriumΔ*spiC* was 10^5^ times higher than that of the parent strain in mice (16). Our research results were similar to these research results. Our result showed that the oral LD_50_ of both SG005Δ*waaJ* and SG005Δ*spiC* was about 10^5^-fold higher than that of the wild strain SG005 (Table 1). In addition, our results showed that when the 7-day-old broilers were administered intramuscularly, SG005Δ*waaJ*Δ*spiC* was the least virulent among the three deletion strains, followed by SG005Δ*spiC* and finally SG005Δ*waaJ* (Table 1). In addition, it should be noted that SG005Δ*waaJ*Δ*spiC* was highly avirulent (IM LD_50_ increased at least by 10^10^-fold) because it did not cause mortality even in response to a high-dose challenge (10^11^ CFU). Furthermore, our results showed that the oral LD_50_ of SG005Δ*waaJ*Δ*spiC* (>10^11^ CFU) was higher than that of SG9R (5 × 10^10^ CFU) (36). Taken together, these results indicated that SG005Δ*waaJ*Δ*spiC* was extremely safe for broilers.

After intramuscular inoculation with SG005Δ*waaJ*Δ*spiC*, the chickens did not show any of the clinical signs, and the weight of the inoculated chickens did not differ from that of the negative control group. This suggested that SG005Δ*waaJ*Δ*spiC* did not cause any obvious damage to the chickens and was quite safe. In the colonization experiment, our results showed that SG005Δ*waaJ*Δ*spiC* could exist in the chicken for at least 10 days and could not be detected on 14 dpi (Figure 4A). Some research reports have pointed out that the temporary residence of bacteria in the internal organs of chickens was essential for inducing protective immunity (37). In addition, SG005Δ*waaJ*Δ*spiC* was cleared faster from the liver and spleen than the two single deletion strains SG005Δ*waaJ* and SG005Δ*spiC* (Figure 4A). In previous experiments using chicken embryos to evaluate the virulence of *S. enterica* ser. Gallinarum, we have reported that the higher was the virulence of the strain, the stronger was its invasiveness into the liver. In this colonization experiment, since SG005Δ*waaJ*Δ*spiC* had the lowest virulence had the weakest invasiveness to chickens, it was eliminated first. According to the above results, it can be interpreted that the inoculation of SG005Δ*waaJ*Δ*spiC* did not cause any clinical signs and had no effect on body weight and that it could colonize in the chicken for at least 10 days.

Generally, the stronger is the virulence of the bacteria, the higher is the immunogenicity. Therefore, the construction of live attenuated *Salmonella* vaccine needs to balance attenuation and immunogenicity. According to the results of the LD_50_ assessment, the virulence reduction of SG005Δ*waaJ*Δ*spiC* was verified, and herein, the protective efficacy was also verified. SG005Δ*waaJ*Δ*spiC* provided protection similar to SG9R (92 vs. 83%, respectively) by intramuscular injection (Figure 4B). More importantly, through oral immunization, the protection of SG005Δ*waaJ*Δ*spiC* could reach 83%, indicating that this double strain can be developed as an oral vaccine. In addition, severe clinical signs of FT, such as ruffled feathers, anorexia, somnolence, and greenish diarrhea, were observed in the PBS group 2 (positive control), whereas no obvious clinical signs were observed in the SG005Δ*waaJ*Δ*spiC* group. For the vaccine strain SG9R, two times of immunization were required, and subcutaneous injection and intramuscular injection were the main ways of vaccination (6). However, for SG005Δ*waaJ*Δ*spiC*, only a single intramuscular injection or oral immunization can achieve an immune effect no less than that of the vaccine strain SG9R. Taken together, these results indicated that attenuated SG005Δ*waaJ*Δ*spiC* could confer effective protection against acute systemic FT infection.

Live attenuated *Salmonella* vaccine can stimulate the body to produce humoral immunity, which is essential for preventing *Salmonella* infection. In this study, we used indirect ELISA to check the serum IgG antibody to assess the level of specific humoral immune response induced by different deletion strains. The results showed that the antibody level of the SG005Δ*waaJ*Δ*spiC* group immunized by intramuscular injection was not significantly different from that of the SG9R group (*P* > 0.05; Figure 4C). This indicated that SG005Δ*waaJ*Δ*spiC* could provide antibody levels comparable to SG9R. A previous similar study reported that *S. enterica* ser. Pullorum S06004Δ*spiC*Δ*waaL* (*waaL*, also a gene involved in the synthesis of LPS) led to a significant antibody increase in broilers (21). In brief, SG005Δ*waaJ*Δ*spiC* induced a strong humoral immune response. The disadvantage of this study was that we did not evaluate the cellular immune responses induced by the deletion strains. The cellular immunity induced by attenuated *Salmonella* is very important in the evaluation of vaccines, because *Salmonella* is an intracellular pathogen. Unfortunately, we did not test this indicator. Since this indicator can only be tested in animal experiments, we plan to test the cellular immune response through cell proliferation experiments in the next animal experiment.

It has been described that some LPS deficient mutants (*waaJ, waaL, rfaH, rfbH, rfbG, et al*.) were used in *Salmonella* DIVA vaccine research (22, 29, 30, 34, 38). It was reported that immunization of piglets with the Δ*waaJ* or Δ*waaL* mutants of *S. enterica* ser. Typhimurium resulted in the induction of a serological response lacking detectable antibodies against LPS, which allowed a clear differentiation between serums from pigs immunized with the Δ*waaJ* or Δ*waaL* strains and serums from pigs infected with the wild type strain (30). There was also a report showing that vaccination of swine with the *rfaH* (a gene encoding the RfaH antiterminator that prevents premature termination of long mRNA transcripts) mutant conferred protection against challenge with virulent *S. enterica* ser. Typhimurium but did not interfere with herd level monitoring for *Salmonella* spp (38). *RfbH* and *rfbG* (both genes are related to LPS biosynthesis) mutants of *S. enterica* ser. Enteritidis and *S. enterica* ser. Pullorum S06004Δ*spiC*Δ*waaL* were also reported to have DIVA features (22, 34). Our results showed that the antibodies produced by SG005Δ*waaJ* and SG005Δ*waaJ*Δ*spiC* did not react with *S. enterica* ser. Pullorum/Gallinarum standard antigen, indicating that these two deletion strains have good DIVA capability (Figure 4D). In addition, the limitations of the DIVA need to be pointed out in the present study. For example, when chickens are infected with a low level of wild strains in the field, the resulting serums may not agglutinate with the standard antigen, because the sensitivity of plate agglutination test is lower than that of ELISA (39). Immunization of animals with these deletion strains will not interfere with the *Salmonella* monitoring program. The vaccine with DIVA capability has the characteristic of distinguishing between infected and immunized animals, and its application to the prevention and control of livestock and poultry diseases is of great significance.

Additionally, although we have verified the deletion strains by PCR and sequencing, to perfect verification of our candidate, we will conduct of characteristics study using southern blot, whole-genome sequencing, immunological study, and field trials.

In this study, we lacked novelty in the selection of target genes, because *waaJ* deletion strains and *spiC* deletion strains have been reported on many other *Salmonella* species (16, 21, 23, 29, 30, 35). However, the *waaJ* and *spiC* double-gene deletion strain has not been reported in *S. enterica* ser. Gallinarum. Another important aspect was that we used a Korean epidemic strain, which was selected through PFGE analysis and antimicrobial sensitivity testing from many clinical isolates. Finally, we want to re-explain the purpose of our experiment. We hope to use the advantages of the commercial vaccine strain (removing the *waaJ* gene to produce DIVA effects and at the same time reduce virulence) and make up for the disadvantages (removing *spiC* to further eliminate residual virulence) to develop a new vaccine based on a Korean epidemic strain of *S. enterica* ser. Gallinarum.

## Conclusion

In summary, we have demonstrated that the rough attenuated *S. enterica* ser. Gallinarum vaccine candidate SG005Δ*waaJ*Δ*spiC* had attributes required in a potent vaccine, such as high attenuation, good immunogenicity, and distinguishable capability. Therefore, this double deletion strain—SG005Δ*waaJ*Δ*spiC*—may be an effective vaccine for controlling *S. enterica* ser. Gallinarum infection in broilers.

## Data Availability Statement

The original contributions presented in the study are included in the article/Supplementary Material, further inquiries can be directed to the corresponding author/s.

## Ethics Statement

The animal study was reviewed and approved by Animal Care and Ethics Committee of Jeonbuk National University.

## Author Contributions

J-FZ, MK, and H-KJ contributed to conception and design of experiments. S-YC, KS, Y-JL, and J-YP contributed to acquisition, analysis, and interpretation of data. J-FZ, MK, S-YC, BW, and H-KJ drafted and/or revised the article. All authors have read and agreed to the published version of the manuscript.

## Funding

This work was supported by Korea Institute of Planning and Evaluation for Technology in Food, Agriculture and Forestry (IPET) through Agriculture, Food and Rural Affairs Convergence Technologies Program for Educating Creative Global Leader (716002-7, 320005-4) funded by Ministry of Agriculture, Food and Rural Affairs (MAFRA). The funders had no role in study design, data collection and analysis, decision to publish, or preparation of the manuscript. We have not received for open access publication fees.

## Conflict of Interest

The authors declare that the research was conducted in the absence of any commercial or financial relationships that could be construed as a potential conflict of interest.

## Publisher's Note

All claims expressed in this article are solely those of the authors and do not necessarily represent those of their affiliated organizations, or those of the publisher, the editors and the reviewers. Any product that may be evaluated in this article, or claim that may be made by its manufacturer, is not guaranteed or endorsed by the publisher.
