# Supplementary material for: Evaluation of Safety and Protective Efficacy of a waaJ and spiC Double Deletion Korean Epidemic Strain of Salmonella enterica Serovar Gallinarum
Source: Front Vet Sci. 2021 Nov 16;8:756123. doi: 10.3389/fvets.2021.756123 (PMC8635151; doi:10.3389/fvets.2021.756123)
Supplement: Supplementary file 1 [file Data_Sheet_1.pdf]

Supplementary Material

1 Supplementary Figure 1

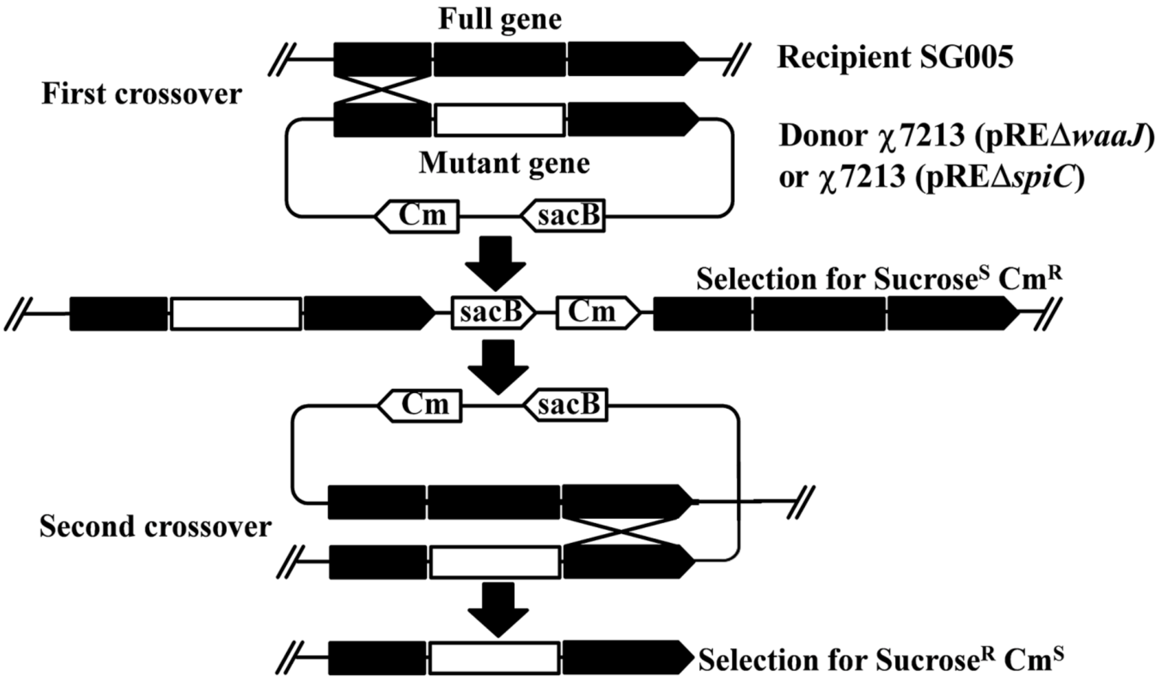

Supplementary Figure 1 | Construction of deletion strains by homologous recombination. The recombinant suicide vector was inserted into the host chromosome by the first crossover, and then the suicide vector with the target gene was deleted from the host chromosome by the second crossover. $Sucrose^S$ Cm^R, sucrose sensitive chloramphenicol resistant; $Sucrose^R$ Cm^S, sucrose resistant chloramphenicol sensitive.

2 Supplementary Figure 2

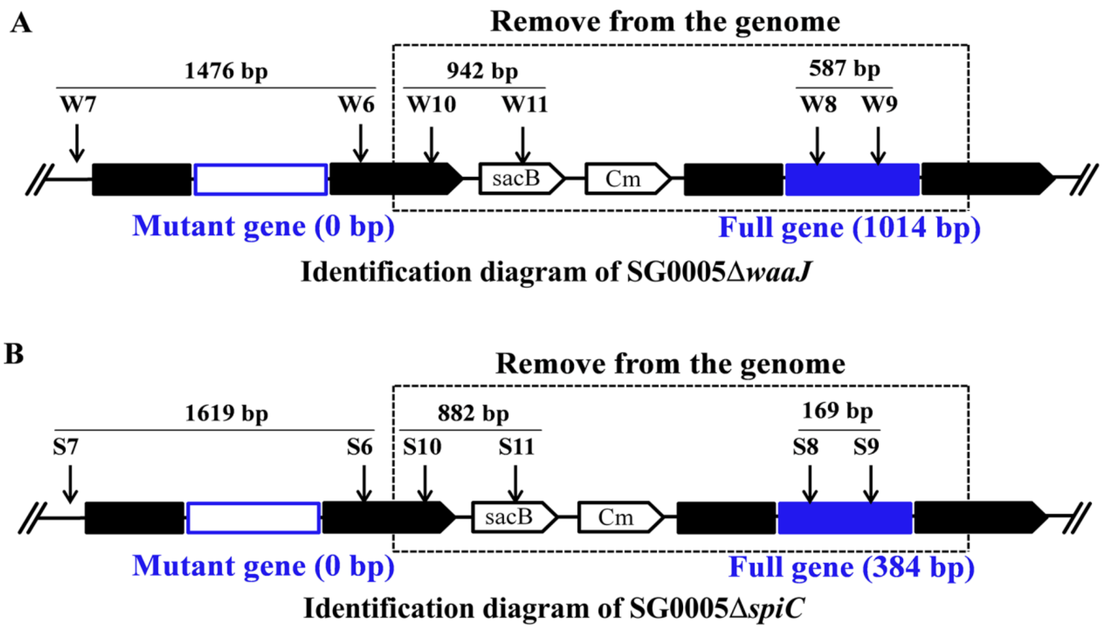

Supplementary Figure 2 | Identification diagrams of deletion strains. (A) Identification diagram of SG0005Δ*waaJ*. W6/W7 was used to confirm the mutant *waaJ* gene. W8/W9 was used to confirm whether the full *waaJ* gene was removed from the genome. W10/W11 was used to confirm whether the suicide plasmid was removed from the genome. (B) Identification diagram of SG0005Δ*spiC*. S6/S7 was used to confirm the mutant *spiC* gene. S8/S9 was used to confirm whether the full *spiC* gene was removed from the genome. W10/W11 was used to confirm whether the suicide plasmid was removed from the genome.

3 Supplementary Figure 3

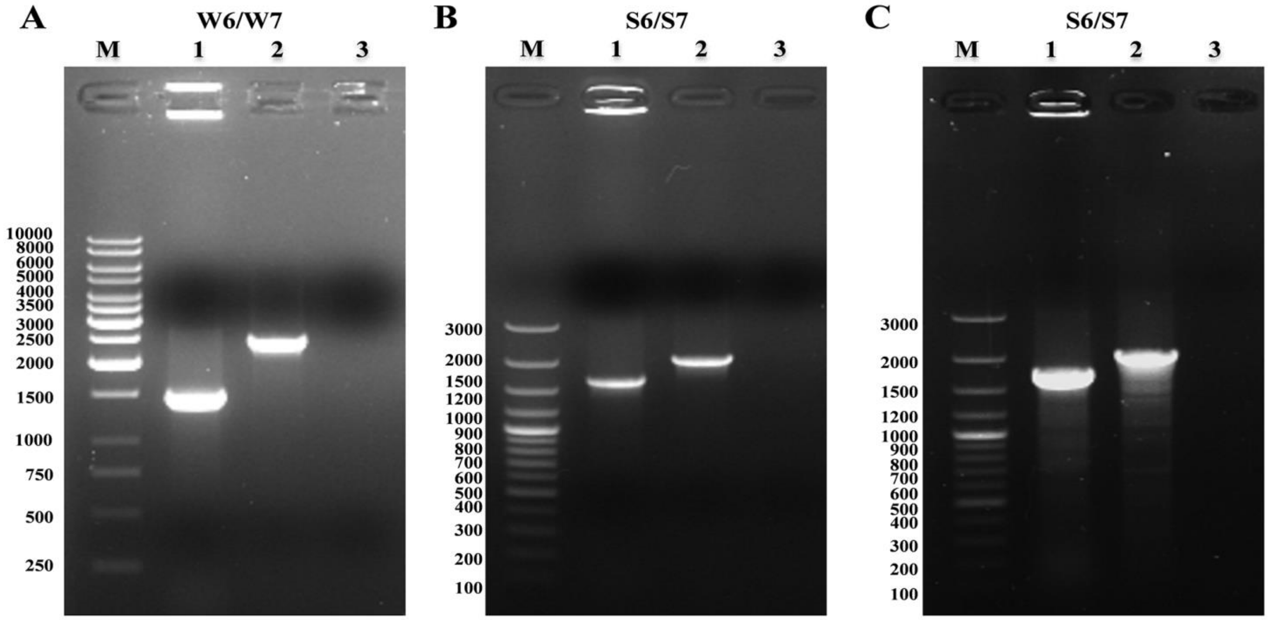

Supplementary Figure 3 | PCR identification of *S. enterica* ser. Gallinarum deletion strains. (A) PCR identification of SG005Δ*waaJ* with W6 and W7. M: DNA Ladder; 1: SG005Δ*waaJ*; 2: SG005; and 3: Negative control. (B) PCR identification of SG005Δ*spiC* with S6 and S7. M: DNA Ladder; 1: SG005Δ*spiC*; 2: SG005; and 3: Negative control. (C) PCR identification of SG005Δ*waaJ*Δ*spiC* with S6 and S7. M: DNA Ladder; 1: SG005Δ*waaJ*Δ*spiC*; 2: SG005Δ*waaJ*; and 3: Negative control.

4 Supplementary Figure 4

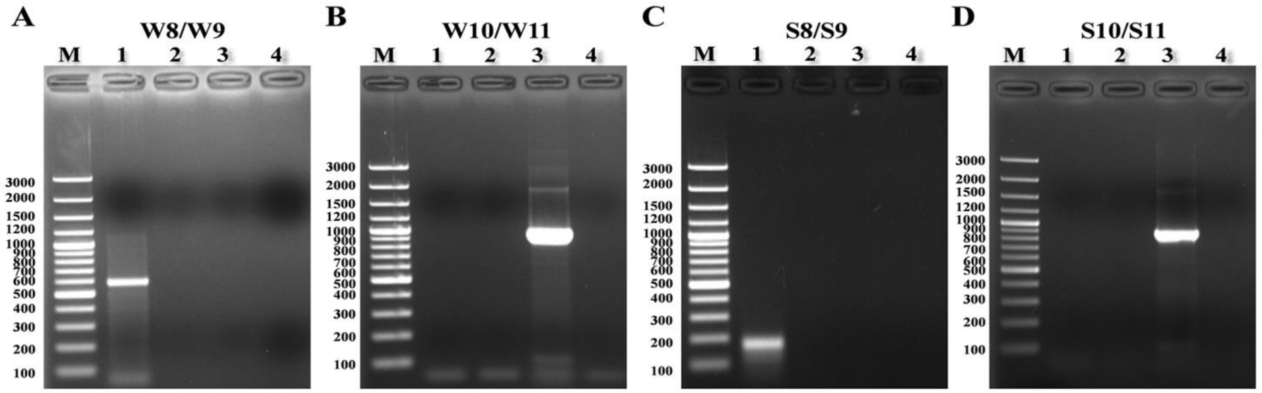

Supplementary Figure 4 | PCR identification of *S. enterica* ser. Gallinarum deletion strains. (A) PCR identification of SG005Δ*waaJ* and SG005Δ*waaJ*Δ*spiC* with W8 and W9. M: DNA Ladder; 1: SG005; 2: SG005Δ*waaJ*; 3: SG005Δ*waaJ*Δ*spiC*; and 4: Negative control. (B) PCR identification of SG005Δ*waaJ* and SG005Δ*waaJ*Δ*spiC* with W10 and W11. M: DNA Ladder; 1: SG005Δ*waaJ*; 2: SG005Δ*waaJ*Δ*spiC*; 3: pREΔ*waaJ* control; and 4: Negative control. (C) PCR identification of SG005Δ*spiC* and SG005Δ*waaJ*Δ*spiC* with S8 and S9. M: DNA Ladder; 1: SG005; 2: SG005Δ*spiC*; 3: SG005Δ*waaJ*Δ*spiC*; and 4: Negative control. (D) PCR identification of SG005Δ*spiC and* SG005Δ*waaJ*Δ*spiC* with S10 and S11. M: DNA Ladder; 1: SG005Δ*spiC*; 2: SG005Δ*waaJ*Δ*spiC*; 3: pREΔ*spiC* control; and 4: Negative control.

5 Supplementary Table 1

Supplementary Table 1 | The primer sequences used for PCR in this study.

Gene amplified	Primers	Sequences[a] (5'-3')	Amplicon size (bp)	Restriction site
Upstream of *waaJ*	W1	GCTCTAGATTCAACAGACTTTAGATTA	1,000	*Xba*I
	W2	TTTGGATCCTACATCACCTATGGGTTTTA		*Bam*HI
Downstream of *waaJ*	W3	TTTGGATCCTTCCCCTGCCTAAATCTCT	1,000	*Bam*HI
	W4	TTTGGTACCTCATTCGTAACAAGGACAGC		*Kpn*I
Upstream of *spiC*	S1	GCTCTAGAAACCGCAACGGAAGCGTGCC	1,000	*Xba*I
	S2	TTTGGATCCGAATCCCTCCTCAGACATAA		*Bam*HI
Downstream *of spiC*	S3	TTTGGATCCAATGGTAGTAAATAAACGTT	1,000	*Bam*HI
	S4	CGAGCTCTTCCAGGGCATTCAAACGCA		*Sac*I
WaaJ (W5/W6) /Δ*waaJ* (W5/W6)	W5	CAGTCACTAGCCATCAGT	Wild strian: 1,589<br>Deletion strain: 575	-
	W6	TCTCACCTCGTCAGAAAT		-
WaaJ (W7/W6) /Δ*waaJ* (W7/W6)	W7	ATGAGCAGAAAATATTTTG	Wild strain: 2,490<br>Deletion strain: 1,476	-
	W6	TCGGGTGCGGTGCTTTACGG		-
WaaJ (W8/W9) /Δ*waaJ* (W8/W9)	W8	TCTTGATGGTGTGGGGGTAT	Wild strain: 587<br>Deletion strain: 0	
	W9	ATGGTCGCGGTAGAAAAATG		
WaaJ (W10/W11) /Δ*waaJ* (W10/W11)	W10	TAACTCACGCCGATGTTCTG	pREΔ*waaJ*: 942<br>Deletion strain: 0	
	W11	TGCTGAACATCAAAGGCAAG		
SpiC (S5/S6) /Δ*spiC* (S5/S6)	S5	ATTCATAATGCTTCCCTC	Wild strain: 969<br>Deletion strain: 585	-
	S6	TAGCCGTATCATAGTTCTC		-
SpiC (S7/S6) /Δ*spiC* (S7/S6)	S7	AGTCGTTGTGCTGGTAAA	Wild strain: 2,003<br>Deletion strain: 1,619	-
	S6	TAGCCGTATCATAGTTCTC		-
SpiC (S8/S9) /Δ*spiC* (S8/S9)	S8	TCAGGATATCAAGGCCGAAG	Wild strain: 169<br>Deletion strain: 0	
	S9	TGCTGCAAGCAGTAGTGTCA		
SpiC (S10/S11) /Δ*spiC* (S10/S11)	S10	GGTGCTAGCGGTTTTTCAAC	PREΔ*spiC*: 882<br>Deletion strain: 0	
	S11	TGCTGAACATCAAAGGCAAG		
pBR322-*waaJ*	pBR322-*waaJ*-F	CGGGATCCTCATGGATTCATTTCCTGAGAT	*waaJ:* 1014	*Bam*HI
	pBR322-*waaJ*-R	GCGTCGACTTATTTGTGGAAAAGTTTAC		*Sal*I
pBR322-*spiC*	pBR322-*spiC*-F	CGGGATCCTCATGCTGGCAGTTTTAAAAGG	*spiC*: 384	*Bam*HI
	pBR322-*spiC*-R	GCGTCGACTTATACCCCACCCGAATAAA		*Sal*I
pBR322-*waaJ-spiC*	pBR322-double-F1	CGGCTAGCGATGGATTCATTTCCTGAGAT	*waaJ:* 1011	*Nhe*I
	pBR322-double-R1	CGGGATCCTTTGTGGAAAAGTTTAC		*Bam*HI
	pBR322-double-F2	CGGGATCCATGCTGGCAGTTTTAAAAGG	*spiC*: 384	*Bam*HI
	pBR322-double-R2	GCGTCGACTTATACCCCACCCGAATAAA		*Sal*I

[a]Underlined sequences represent restriction enzyme recognition sites.

6 Supplementary Table 2

Supplementary Table 2 | Analysis of biochemical properties for the wild-type *S. enterica* ser. Gallinarum and the deletion strains

Biochemicals	SG005	SG005Δ*waaJ*	SG005Δ*spiC*	SG005Δ*waaJ*Δ*spiC*
Acetoin production	-	-	-	-
Amygdalin fermentation	-	-	-	-
Arabinose fermentation	-	-	-	-
Arginine dihydrolase	-	-	-	-
Beta-galactosidase	-	-	-	-
Citrate utilization	-	-	-	-
Gelatinase	-	-	-	-
Glucose fermentation	+	+	+	+
H_2S production	-	-	-	-
Indole production	-	-	-	-
Inositol fermentation	-	-	-	-
Lysine decarboxylase	+	+	+	+
Mannitol fermentation	+	+	+	+
Melibiose fermentation	-	-	-	-
Ornithine decarboxylase	-	-	-	-
Rhamnose fermentation	-	-	-	-
Sorbitol fermentation	-	-	-	-
Sucrose fermentation	-	-	-	-
Tryptophane deaminase	-	-	-	-
Urease	-	-	-	-

7 Supplementary Text 1 | Sequence results of the deletion strains.

7.1 Sequencing result of SG005Δ*waaJ*

ATGAGCAGAAAATATTTTGAAGAAGAAGTCATTCAACAGACTTTAGATTATAACTATGC
ACAACATAGTGATGCTGCTAAATTTAATATAGCTTATGGGATTGATAAAAACTTTCTTTT
TGGCTGTGGTGTCTCTATTGCATCGGTTCTCCTCGCTAACCCAGAGAAGGCGTTAGCTTT
CCATGTTTTTACCGATTTCTTTGGCTCTGAAGACCAGCAGCGATTTGAGGCATTAGCAAA
ACAGTACGCTACGCAGATTGTTGTTTACCTAATCGACTGTGAGCGCTTAAAATCGTTACC
CAGTACCAAAAACTGGACCTATGCAACATACTTTAGATTCATTATCGCCGATTATTTTTC
AGATAAAACAGATAGAGTACTTTATCTGGATGCAGATATTGCATGTAAGGGGAGTATTC
AGGAACTTATTGATCTTAATTTTGCTGAAAATGAGATTGCGGCTGTCGTTGCTGAAGGCG
AGTTGGAATGGTGGACTAAGCGCTCGGTTAGCCTGGCAACGCCTGGGCTGGTTTCTGGC
TATTTTAATGCCGGTTTTATTTTAATTAACATACCTCTTTGGACCGCAGAAAATATCTCTA
AGAAAGCGATTGAAATGCTAAAAGATCCAGAGGTAGTACAGCGCATAACGCACCTTGA
TCAGGATGTATTAAATATATTGTTAGTGAATAAAGCGCGTTTTGTTGATAAAAAGTTTAA
TACACAATTTAGTCTTAACTATGAATTAAAAGATTCAGTTATTAATCCAGTCGATGCTGA
TACTGTATTTGTTCATTATATCGGACCAACGAAGCCCTGGCATAGTTGGGGGGCTTACCC
TGTGTCACAATATTTTTTACAGGCTAAGAGCAACTCACCGTGGTCTCATTGTGCACTTTT
AAATCCAGTCACTAGCCATCAGTTACGTTATGCGGCAAAGCATATGTTTAATCAGAAGC
ATTATACTTCGGGTATAAATTACTATATAGCCTACTTTAAACGTAAACTTCTTGAATAAA
ACCCATAGGTGATGTAGGATCCTTCCCCTGCCTAAATCTCTATTATGATTATTGAAAAAA
AGATTAAAAACTATACCGTCTTTGTCAAAAAAGACGGTGAAAAATACATTGAGATATTC
AAAGATTTTCTATCTTATAATCATCAGGTTATTAAAGTCTTCCGTAATATAGAAGATACA
AAAGTTGTATTGATTAATACAGACTACGGAAAATATATTCTCAAGGTATTTAGTCCAAA
AGTAAAAAATACTGAACGATTTTTCAAATCGTTAGTAAAAGGAGATTACTACGAAAAGC
TTTTTCATCAGACAGATCGTGTTCGGCGAGAAGGGTTTACGGCGCTTAATGATTTTTACC
TGCTGGCGGAAATTAAAACATTACGCTATGTAAAAACATACGTGATGATAATTGAGTAT
ATTGAAGGTATTGAACTTGTTGATATGCCAGAAATTTCTGACGAGGTGAGA

Blue part: *waaJ* upstream gene; Green part: Non-coding sequence; Pink part: *waaJ* downstream gene partial sequence; Red part: *Bam*HI(The *waaJ* gene was completely removed, leaving a restriction site *Bam*HI).

7.2 Sequencing result of SG005Δ*spiC*

TTGGCCAGTGAGCGATGTAGTAACCAGCAAAAAGCCGACGTCATCAACACCAATGCTGT
TAATGTAAAGGGGATTTGTTGAAGGATAATTTTTAAGATGCGATTATGTAGATTACCGTA
TGGGTACAGCGTAACCAGACTCCATCCGGGGCCATGCAAGGTTGTGCGTAATATCAGAA
ATCCGGGAATTTGCTGCCATCCATCATGCAGCGTTACATTTTCTAACTGTGTACGTATTTT
TTGCGGGATGTATGAAAACGGCAATAAGTGGTTGTTTTGATCCAGCCATACTCGAATAC
TATCATCTAATGGCAGGTGGCTCTTAGTAATGAGATCGGGAAGTTTAACCGTCACCCCA
AAAAATACGCCTTGCTGATCGGCAACCGCAACGGAAGCGTGCCATCCTTTGCCGTTTAT
GTATTCTGGTTCACTCCAGTAAAACCCGGCATGGGTTGGGTATAAAGGAAAGCTTTTTCG
CGTTAAAGGCTGTAGAGTTGAATAATCTGAAGGGTTATCAGTAGATAATAACGAAATCT
CATTTTTATGATTAAGAATAAAACTATCGCGACGAAAGCTATTTTCAACGATATCAGAG

GACTGCAGAAAGAGACGGTGCTTCTCTCCGTTTAGCGTCGGCGTGCAATTTGAAGGACC
GACAGATAGATGCCGGCTCACCTCAGGGAAAATATCGTTATGATGAATCTCAGTCGCTA
ATGAGCATTGATACATTAAATTTTTAGCGTCACGTTCAGCTTCTTCAAACCGTTGATTGC
TTAGTACAATATTCATCTCGGATAGAACGGATAAATCCTCTATTATATGCTGCCGTTTCT
GAACCATTGATATATAAGCTGCGGTAAGCACAGATAGCAGCCAAATAATTATTGTTGTT
AATAAAAATAAAAAAGTTAGCCTGATTACTAAAGATGTTTGCAGCGTATTCTTGAGATT
GAGCAAATTCATAATGCTTCCCTCCAGTTGCCTGTTGCAAAATCTTTGGCACTTGATCAC
TATCGCAGTACATATAGTTTCATCAGAAGATTAATCGATGGTGTTATCATTAGGAAGATA
AATTTCTTCATATATAACCCAGTCGATGACTACAATTACTTTTTAATAAGATGGCGATGT
AAAAACATCGTAACAGTTCATTTAATAAATGATTTTTCAAATTGTAAGTTTTTATGTCAG
TGCTGAAAATGTAATTGTGAATTTATCGGAAAATCCGAATGATAGAATCGCCTGTGACA
AGGTATATGTAGACAGCATCCTGATATTGTACAAGAAAAGTATAGTCGAAATAAATGTG
AATCAGGCTTTTTACGGATGTGGTTGTGAGCGAATTTGATAGAAACTCCCATTTATGTCT
GAGGAGGGATTCGGATCCAATGGTAGTAAATAAACGTTTAATCTTAATTTTACTATTTAT
ACTCAATACAGCAAAGAGTGATGAGTTATCATGGAAAGGTAATGACTTCACCCTTTATG
CCAGACAAATGCCATTAGCAGAGGTTTTACATCTGCTCTCAGAGAACTATGATACGGCT
A

Blue part: *spiC* upstream gene partial sequence; Green part: Non-coding sequence; Pink part: *spiC* downstream gene partial sequence; Red part: *Bam*HI(The *spiC* gene was completely removed, leaving a restriction site *Bam*HI).

7.3 Sequencing result of SG005Δ*waaJ*Δ*spiC*

TTGGCCAGTGAGCGATGTAGTAACCAGCAAAAAGCCGACGTCATCAACACCAATGCTGT
TAATGTAAAGGGGATTTGTTGAAGGATAATTTTTAAGATGCGATTATGTAGATTACCGTA
TGGGTACAGCGTAACCAGACTCCATCCGGGGCCATGCAAGGTTGTGCGTAATATCAGAA
ATCCGGGAATTTGCTGCCATCCATCATGCAGCGTTACATTTTCTAACTGTGTACGTATTTT
TTGCGGGATGTATGAAAACGGCAATAAGTGGTTGTTTTGATCCAGCCATACTCGAATAC
TATCATCTAATGGCAGGTGGCTCTTAGTAATGAGATCGGGAAGTTTAACCGTCACCCCA
AAAAATACGCCTTGCTGATCGGCAACCGCAACGGAAGCGTGCCATCCTTTGCCGTTTAT
GTATTCTGGTTCACTCCAGTAAAACCCGGCATGGGTTGGGTATAAAGGAAAGCTTTTTCG
CGTTAAAGGCTGTAGAGTTGAATAATCTGAAGGGTTATCAGTAGATAATAACGAAATCT
CATTTTTATGATTAAGAATAAAACTATCGCGACGAAAGCTATTTTCATCGATATCAGAGG
ACTGCAGAAAGAGACGGTGCTTCTCTCCGTTTAGCGTCGGCGTGCAATTTGAAGGACCG
ACAGATAGATGCCGGCTCACCTCAGGGAAAATATCGTTATGATGAATCTCAGTCGCTAA
TGAGCATTGATACATTAAATTTTTAGCGTCACGTTCAGCTTCTTCAAACCGTTGATTGCTT
AGTACAATATTCATCTCGGATAGAACGGATAAATCCTCTATTATATGCTGCCGTTTCTGA
ACCATTGATATATAAGCTGCGGTAAGCACAGATAGCAGCCAAATAATTATTGTTGTTAA
TAAAAATAAAAAAGTTAGCCTGATTACTAAAGATGTTTGCAGCGTATTCTTGAGATTGA
GCAAATTCATAATGCTTCCCTCCAGTTGCCTGTTGCAAAATCTTTGGCACTTGATCACTA
TCGCAGTACATATAGTTTCATCAGAAGATTAATCGATGGTGTTATCATTAGGAAGATAA
ATTTCTTCATATATAACCCAGTCGATGACTACAATTACTTTTTAATAAGATGGCGATGTA
AAAACATCGTAACAGTTCATTTAATAAATGATTTTTCAAATTGTAAGTTTTTATGTCAGT
GCTGAAAATGTAATTGTGAATTTATCGGAAAATCCGAATGATAGAATCGCCTGTGACAA
GGTATATGTAGACAGCATCCTGATATTGTACAAGAAAAGTATAGTCGAAATAAATGTGA

ATCAGGCTTTTTACGGATGTGGTTGTGAGCGAATTTGATAGAAACTCCCATTTATGTCTG
AGGAGGGATTCGGATCCAATGGTAGTAAATAAACGTTTAATCTTAATTTTACTATTTATA
CTCAATACAGCAAAGAGTGATGAGTTATCATGGAAAGGTAATGACTTCACCCTTTATGC
CAGACAAATGCCATTAGCAGAGGTTTTACATCCGCTCTCAGAGAACTATGATACGGCT

Blue part: *spiC* upstream gene partial sequence; Green part: Non-coding sequence; Pink part: *spiC* downstream gene partial sequence; Red part: *Bam*HI(The *spiC* gene was completely removed, leaving a restriction site *Bam*HI).
